# Apollon gene silencing induces apoptosis in breast cancer cells through p53 stabilisation and caspase-3 activation

**DOI:** 10.1038/sj.bjc.6604927

**Published:** 2009-02-17

**Authors:** A Lopergolo, M Pennati, P Gandellini, N I Orlotti, P Poma, M G Daidone, M Folini, N Zaffaroni

**Affiliations:** 1 Department of Experimental Oncology, Fondazione IRCCS Istituto Nazionale dei Tumori, via Venezian 1, 20133 Milano, Italy; 2 Department of Pharmacological Sciences, University of Palermo, via del Vespro 129, 90127 Palermo, Italy

**Keywords:** Apollon, human breast cancer, siRNA, apoptosis, p53, caspase-3

## Abstract

We analysed the effects of small interfering RNA (siRNA)-mediated silencing of Apollon, a member of the inhibitors of apoptosis protein family, on the proliferative potential and ability of human breast cancer cell lines to undergo apoptosis. In wild-type p53 ZR75.1 cells, Apollon knockdown resulted in a marked, time-dependent decline of cell growth and an increased rate of apoptosis, which was associated with p53 stabilisation and activation of the mitochondrial-dependent apoptotic pathway. Pre-incubation of cells with a p53-specific siRNA resulted in a partial rescue of cell growth inhibition, as well as in a marked reduction of the apoptotic response, indicating p53 as a major player in cell growth impairment consequent on Apollon silencing. Apollon knockdown induced consistently less pronounced anti-proliferative and pro-apoptotic effects in mutant p53 MDA-MB-231 cells than in ZR75.1 cells. Furthermore, the activation of caspase-3 seemed to be essential for the induction of apoptosis after Apollon knockdown, as the Apollon-specific siRNA had no effect on the viability of caspase-3-deficient, wild-type p53 MCF-7 cells or the ZR75.1 cells after RNA interference-mediated caspase-3 silencing. Our results indicate that p53 stabilisation and caspase-3 activation concur to determine the apoptotic response mediated by Apollon knockdown in breast cancer cells, and suggest Apollon to be a potential new therapeutic target for this malignancy.

Apoptosis is a tightly regulated process, which plays a central role in the development and homeostasis of multicellular organisms. Its deregulation is involved in a wide spectrum of diseases including cancer, in which an overexpression of anti-apoptotic proteins endows cells with a selective survival advantage that promotes malignancy (Green and Evan, 2002). The members of the inhibitors of apoptosis protein (IAP) family have been proved to be crucial negative regulators of apoptosis (Srinivasula and Ashwell, 2008), and some of these proteins were found to be expressed highly in human tumours (Hong *et al*, 2003; Yang *et al*, 2003; Srinivasula and Ashwell, 2008).

Apollon, also known as Bruce or BIRC6, is the largest member of the IAP family, containing one baculoviral IAP repeat domain at its N-terminal region and a C-terminal E2 motif, which can form thioester bonds with ubiquitin (Chen *et al*, 1999). It has also been suggested to function as an E3 ligase, and is itself regulated by ubiquitin-dependent degradation mediated by E2 UbcH5 and E3 Nrdp1 (Qiu and Goldberg, 2005). Although the physiological role of Apollon in counteracting apoptosis is largely unknown, the available information indicates that Apollon exerts its cytoprotective activity by promoting ubiquitination and degradation of the pro-apoptotic protein Smac/DIABLO and by inhibiting caspase activity (Hao *et al*, 2004; Qiu and Goldberg, 2005). It has been reported that Apollon is upregulated in some brain tumour cell lines that are resistant to certain DNA-damaging agents (Chen *et al*, 1999), and also that its overexpression is associated with poor prognosis in childhood acute myeloid leukaemia (Sung *et al*, 2007). These findings, together with preliminary evidence concerning the possibility of sensitising tumour cells to apoptosis induced by some anticancer drugs through antisense oligonucleotide- or small interfering RNA (siRNA)-mediated downregulation of Apollon (Chen *et al*, 1999; Qiu *et al*, 2004; Chu *et al*, 2008), has led researchers to consider the gene as a possible new therapeutic target.

With the aim to elucidate the molecular bases responsible for the cytoprotective activity of Apollon in breast cancer, we investigated the effects of its knockdown, accomplished through RNA interference (RNAi), on the proliferative potential and its ability to undergo apoptosis of established cell lines differing in the *TP53* gene status. The results of this study indicate that wild-type p53 stabilisation and caspase-3 activation concur in determining the apoptotic response, consequent on Apollon knockdown in breast cancer cells.

## Materials and methods

### Cell lines

We used three human breast carcinoma cell lines: ZR75.1 and the caspase-3-deficient MCF-7 cell lines expressing wild-type p53, and the MDA-MB-231 cell line expressing a mutant p53 (Sheikh *et al*, 1993; Janicke *et al*, 1998; Yang *et al*, 1998; Gartel *et al*, 2003) (American Type Culture Collection, Rockville, MD, USA). The ZR75.1 and MCF-7 cells were grown in the RPMI-1640 medium (Lonza Milano s.r.l, Treviglio, Italy) supplemented with 10% foetal bovine serum; the MDA-MB-231 cells were cultured using the D-MEM/F12 medium (Lonza Milano s.r.l) supplemented with 5% foetal bovine serum. Cells were maintained as a monolayer in a humidified incubator at 37°C with a supply of 5% CO_2_ : 95% air atmosphere.

### Design and synthesis of small interfering RNAs

Four different siRNAs targeting specific consensus sequences (5′-AA(N19)-3′), within the open reading frame of Apollon mRNA (GeneBank accession no. NM_016252), were designed using a siRNA target finder tool (http://www.ambion.com) (Table 1). A BLAST search (http://www.ncbi.nlm.nih.gov/BLAST) for selected siRNA sequences was carried out to exclude any alignment with other sequences in the human genome. A control siRNA made up of a scrambled sequence with no significant homology to any known human mRNA was included in this study (Table 1). Small interfering RNAs were manufactured by the Eurofins MWG Operon (Ebersberg, Germany) as preformed and purified duplexes.

### Transfection procedures

The day before transfection, breast cancer cells were seeded at a density of 2 × 10^5^ per 25-cm^2^ flask. A given amount of each Apollon and control siRNA was mixed with Lipofectamine2000™ (Invitrogen, San Giuliano Milanese, Italy) for 20 min at room temperature. The mixtures were then applied to the cells in a volume of Opti-MEM I (Invitrogen), giving a final concentration of 10 nM for each siRNA. After a 4-h incubation at 37°C, cells were washed with PBS and a culture medium supplemented with serum was added. At different intervals after transfection (from 24 to 96 h), cells were collected by trypsinisation and used subsequently in the different assays. In cotransfection experiments, cells were treated with 25 nM caspase-3-specific siRNA or p53-specific siRNA (Santa Cruz Biotechnology, Santa Cruz, CA, USA), cultured for 24 h at 37°C, and then transfected with 10 nM Apollon or control siRNA as described earlier. Cells exposed to Lipofectamine2000 alone were referred to as mock control throughout this study.

### Protein extraction and western blot analysis

Western blots were carried out for the following proteins: apoptosis inducing factor (AIF), Apollon, Bad, Bax, cIAP1, cIAP2, cytochrome *c*, p53, Smac/DIABLO, survivin and XIAP.

Cells were lysed, and mitochondrial and cytosolic fractions were obtained using a mitochondrial/cytosol fractionation kit (Medical & Biological Laboratories, Naka-ku Nagoya, Japan). Samples containing 40 *μ*g of protein per lane were separated on precast 3–8% NuPAGE™ tris-acetate (for the detection of Apollon) or 12% NuPAGE bis-tris (for the detection of AIF, Bad, Bax, cIAP1, cIAP2, cytochrome *c*, p53, Smac/DIABLO, survivin and XIAP) gels (Invitrogen), and were transferred onto Hybond ECL nitrocellulose membranes (GE Healthcare Europe GmbH, Cologno Monzese, Italy) using the NuPAGE transfer buffer (Invitrogen). Nitrocellulose membranes were blocked in PBS-Tween 20 with 5% skim milk, first incubated with the primary antibodies (Abcam Inc., Cambridge, MA, USA) and then with the secondary peroxidase-linked whole antibodies (GE Healthcare Europe). Bound antibodies were detected using the SuperSignal® West PICO chemiluminescent substrate (Pierce Biotechnology Inc., Rockford, IL, USA). *β*-Actin and COX IV monoclonal antibodies (Abcam) were used to confirm equal protein loading on the gel, and also to show the relative purity of the cytosolic or mitochondrial fractions. Filters were autoradiographed, and autoradiographs were scanned and quantified by densitometric analysis.

### Cell growth inhibition assay and apoptosis analysis

At different intervals after transfection with siRNAs, adherent cells were trypsinised and counted in a particle counter (Coulter Counter, Coulter Electronics, Luton, UK). The cell viability was determined by the Trypan blue dye exclusion test. Each experimental sample was run in triplicate.

For apoptosis analysis, adherent cells were pooled together with detached cells and then scored for nuclear morphology of apoptosis (chromatin condensation and DNA fragmentation) by labelling with a solution containing 50 *μ*g ml^−1^ of propidium iodide, 50 mg ml^−1^ of RNase and 0.05% Nonidet P40 in PBS. After staining, the slides were examined using fluorescence microscopy, and the percentage of cells with an apoptotic nuclear morphology was determined by scoring at least 500 cells in each sample. In the same cellular samples, the catalytic activity of caspase-9, caspase-3 and caspase-8 was measured as the ability to cleave the specific substrates *N*-acetyl-Leu-Glu-His-Asp-AMC (LEHD-AMC), *N*-acetyl-Asp-Glu-Val-Asp-AMC (DEVD-AMC) and *N*-acetyl-Ile-Glu-Thr-Asp-AMC (IETD-AMC) by means of the APOPCYTO/caspase-9, APOPCYTO/caspase-3 and APOPCYTO/caspase-8 assay kits (Medical & Biological Laboratories), respectively. The hydrolysis of the specific substrates for the different caspases was monitored by spectrofluorometry with 380-nm excitation and 460-nm emission filters.

### Quantification of cytochrome *c* release

The cytochrome *c* release was measured using the Cytochrome *c* ELISA kit (Medical & Biological Laboratories). After colour development had stopped, the absorbance at 450 nm was measured on the microplate reader. Percent release of cytochrome *c* was calculated as the amount of cytosolic cytochrome *c* divided by the total amount of cytosolic and mitochondrial cytochrome *c*.

### Total RNA isolation and RT–PCR

Total RNA isolated using Qiagen RNeasy Mini Kit (Qiagen, Milan, Italy) was reverse-transcribed in the presence of random hexamers using the GeneAmp RNA Core Kit (Applied Biosystems, Foster City, CA, USA). To assess the p53 mRNA expression, the resultant cDNA was amplified through optimised PCR cycling conditions in the presence of specific primer pair (Maxwell *et al*, 1999): the sense primer was 5′-TCTTTGCATTCTGGGACAGCC-3′ and the antisense primer was 5′-AGCTCGTGGTGAGGCTCCCCT-3′ (Eurofins MWG Operon). A fragment corresponding to *β*-actin was used as the standard of the amplification reaction. The PCR products were verified by gel electrophoresis, and the images were acquired by a ScanJET IIcx/T scanner (Hewlett Packard, Milano, Italy).

### Statistical analysis

Student's *t*-test was used to analyse the differences between control and siRNA-transfected cells in terms of protein expression, cell growth, rate of apoptosis, *in vitro* catalytic activity of caspase-9, caspase-3 and caspase-8, and release of cytochrome *c*. *P*-values <0.05 (two-sided) were considered statistically significant.

## Results

### siRNA-mediated Apollon knockdown affects cell growth in breast cancer cells

**Figure 1:**
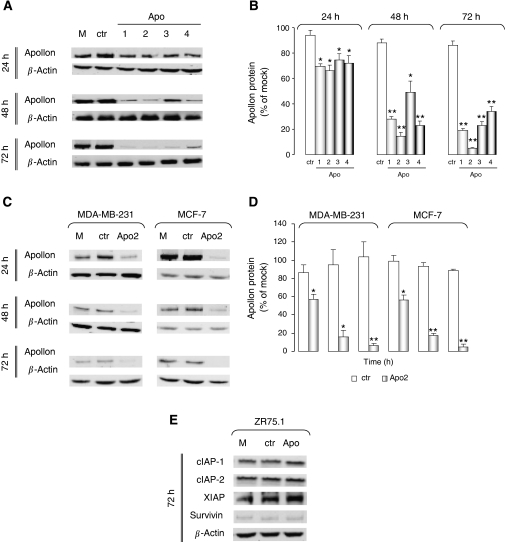
Downregulation of Apollon by siRNA in breast cancer cells. (**A**) A representative western blot experiment showing Apollon protein expression levels in ZR75.1 cells exposed to Lipofectamine2000™ alone (mock control, M) or transfected with 10 nM control (ctr) and Apollon (1–4) siRNAs at various time points after transfection. (**B**) Quantification of the Apollon protein expression in ZR75.1 cells. Data are reported as the percentage of the Apollon expression in cells transfected with control or Apollon-specific siRNAs compared with mock control and represent the mean values±s.d. of at least three independent experiments. ^*^P<0.01 and ^**^P<0.001 *vs* mock control. (**C**) A representative western blot experiment showing Apollon protein expression levels in MDA-MB-231 and MCF-7 cells exposed to mock control (M) or transfected with ctr and Apo2 siRNAs at various time points after transfection. (**D**) Quantification of the Apollon protein expression in MDA-MB-231 and MCF-7 cells. Data are reported as the percentage of Apollon expression in cells transfected with ctr (empty column) or Apo2 (grey column) siRNAs compared with mock control and represent the mean values±s.d. of at least three independent experiments. ^*^P<0.01 and ^**^P<0.001 *vs* mock control. (**E**) A representative western blot experiment showing the expression of other anti-apoptotic proteins belonging to the IAP family in ZR75.1 cells 72 h after transfection with ctr and Apo2 siRNAs. IAP=inhibitors of apoptosis protein; siRNA=small interfering RNA.

To gain insight into the role of Apollon in breast cancer cell survival, we used an RNAi-based strategy to downregulate its expression in three human breast cancer cell lines, characterised by a different *TP53* gene status: ZR75.1 and MCF-7 cells bearing wild-type p53 and MDA-MB-231 cells carrying mutant p53. We first tested the effectiveness of four 21-mer siRNAs targeting different portions within the Apollon mRNA (Table 1), to silence the Apollon gene expression in the ZR75.1 cell line. Western blotting experiments carried out in cells collected at different intervals (24–72 h), after a 4-h transfection with 10 nM of each Apollon-specific siRNA, showed a variable degree of protein expression inhibition as a function of the different oligomer used (Figure 1A and B). Specifically, the abundance of Apollon protein was reduced significantly starting from 24 h after transfection with every siRNA as compared with that in mock control (Figure 1A and B). The extent of the inhibition increased over time and reached its maximum at 72 h after transfection with all siRNAs (Figure 1A and B). Transfection with the Apollon-specific siRNA (Apo2), which was able to induce the greatest inhibition of Apollon expression in the ZR75.1 cell line, also resulted in a significant and time-dependent decline of the protein in the MDA-MB-231 and MCF-7 cell lines (Figure 1C and D). Conversely, Apo2 did not modify the expression of other anti-apoptotic proteins belonging to the IAP family, including cIAP1, cIAP2, XIAP and survivin (Figure 1E).

**Figure 2:**
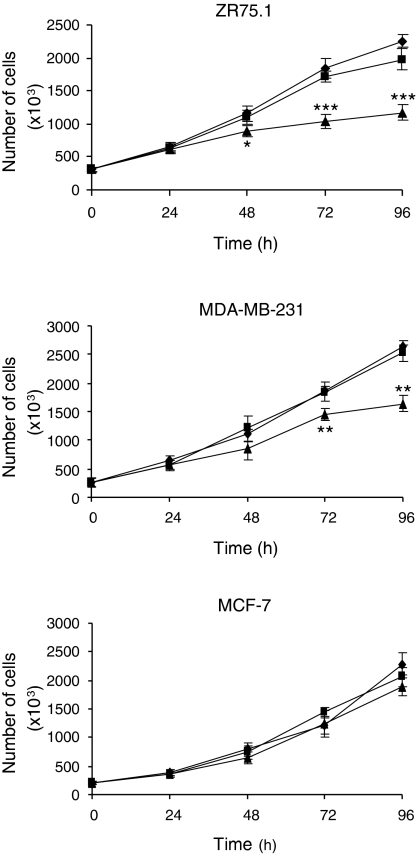
Effects of Apollon downregulation on *in vitro* growth of breast cancer cells. Survival curves of ZR75.1, MDA-MB-231 and MCF-7 cells exposed to mock control (♦) or transfected with ctr (▪) and Apo2 (▴) siRNAs. Points represent the mean values±s.d. of at least three independent experiments. ^*^P<0.05, ^**^P<0.01 and ^***^P<0.001 *vs* mock control.

The effects of Apollon downregulation on the *in vitro* proliferative potential of breast cancer cells were further evaluated using Apo2, which was able to inhibit the protein expression by ∼90% at 72 h after transfection in all cell lines (Figure 1B and D). In ZR75.1 cells, inhibition of Apollon resulted in a significant and time-dependent decrease in viable cell number as compared with that in mock control (Figure 2, upper panel). Such a growth inhibition was appreciable starting 48 h after transfection and increasing progressively over time. Although to a lesser extent, cell growth was affected by Apollon knockdown also in the MDA-MB-231 cells, and a significant reduction in cell number compared with that in mock control was appreciable at 72 and 96 h after transfection (Figure 2, middle panel). Conversely, Apollon downregulation failed to affect the growth of MCF-7 cells at any time point considered (Figure 2, lower panel).

### Apollon knockdown induces apoptosis in breast cancer cells

**Figure 3:**
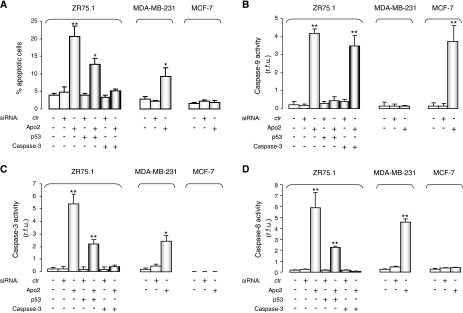
Effects of Apollon downregulation on the apoptotic rate of breast cancer cells. (**A**) The percentage of cells with an apoptotic nuclear morphology as assessed by fluorescence microscopy in ZR75.1, MDA-MB-231 and MCF-7 cells 72 h after transfection with siRNAs. Data represent the mean values±s.d. of at least three independent experiments. ^*^P<0.01 and ^**^P<0.001 *vs* mock control. (**B**) Caspase-9, (**C**) caspase-3 and (**D**) caspase-8 catalytic activity as determined by hydrolysis of specific fluorogenic substrates 72 h after transfection with siRNAs. Data are expressed as relative fluorescence units and represent the mean values±s.d. of at least three independent experiments. ^*^P<0.01 and ^**^P<0.001 *vs* mock control. siRNA=small interfering RNA.

To investigate whether cell growth inhibition consequent on Apollon knockdown was ascribable to the induction of apoptosis, cells were stained with propidium iodide 72 h after transfection, and the presence of cells with an apoptotic nuclear morphology was assessed by fluorescence microscopy. In all cell lines, apoptosis was observed in a modest fraction of cells transfected with the control siRNA (from 2.3 to 4.8% of the overall cell population) and in mock control cells (from 1.7 to 3.8%). Conversely, after the exposure of ZR75.1 cells to Apo2, the percentage of apoptotic cells was about six-fold higher than that of mock control (21.7 *vs* 3.8%) (Figure 3A). A significant, although considerably less pronounced, increase in the percentage of apoptotic cells was also observed in the MDA-MB-231 cells transfected with Apo2 compared with that in mock control (9.3 *vs* 2.9%) (Figure 3A). Consistent with a negligible effect on cell growth, in MCF-7 cells, Apollon knockdown did not induce any increase in the apoptotic cell rate (Figure 3A).

Caspase catalytic activity was further assessed in all cell lines 72 h after transfection. The results revealed that in ZR75.1 cells, Apollon knockdown induced a 20-, 25- and 33-fold increase in caspase-9, caspase-3 and caspase-8 catalytic activity, respectively, compared with that in mock control (Figure 3B–D). A 10- and 17-fold increase in caspase-3 and caspase-8 catalytic activity, respectively, was also observed in MDA-MB-231 cells after exposure to Apo2 (Figure 3C and D), although no appreciable effect on caspase-9 activity was recorded in this cell line (Figure 3B). In MCF-7 cells, exposure to Apo2 induced a 17-fold increase in caspase-9 catalytic activity compared with that in mock control. In agreement with the lack of functional caspase-3 in this cellular model (Janicke *et al*, 1998; Yang *et al*, 1998), no enzyme activity was detectable under any of the treatment conditions (Figure 3C). Moreover, no appreciable activation of caspase-8 was observed in this cell line (Figure 3D).

**Figure 4:**
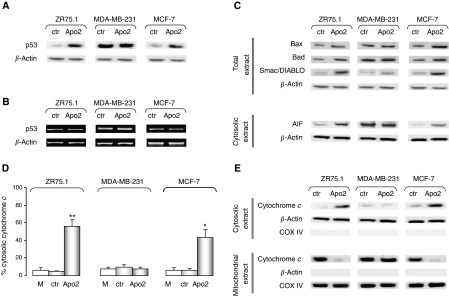
Effects of Apollon downregulation on mitochondrial apoptotic pathway. Representative (**A**) western blot and (**B**) RT–PCR experiments showing the expression of p53 protein and mRNA, respectively, in ZR75.1, MDA-MB-231 and MCF-7 cells 72 h after transfection with ctr and Apo2 siRNAs. (**C**) A representative western blot experiment showing the expression of proteins involved in the mitochondrial pathway, in ZR75.1, MDA-MB-231 and MCF-7 cells 72 h after transfection with ctr and Apo2 siRNAs. (**D**) Cytochrome *c* release as determined by ELISA in ZR75.1, MDA-MB-231 and MCF-7 cells 72 h exposed to mock control or transfected with ctr and Apo2 siRNAs. Data are expressed as percentage values of cytosolic cytochrome *c* with respect to the total amount of cytosolic and mitochondrial cytochrome *c*, and represent the mean values±s.d. of at least three independent experiments. ^*^P<0.01 and ^**^P<0.001 *vs* mock control. (**E**) A representative western blot experiment showing the abundance of cytochrome *c* in mitochondria and cytosolic fractions in ZR75.1, MDA-MB-231 and MCF-7 cells 72 h after transfection with ctr and Apo2 siRNAs. *β*-Actin and COX IV were used to confirm equal protein loading and to show the relative purity of the cytosolic or mitochondrial fractions. AIF=apoptosis inducing factor; siRNA=small interfering RNA.

The activation of caspase-9 in ZR75.1 and MCF-7 cells suggested that the intrinsic (mitochondrial) pathway of apoptosis is activated by Apollon knockdown only in breast cancer cells bearing wild-type p53. To confirm this, we assessed by western blot the total amount of p53 after Apollon silencing in the different cell lines (Figure 4A). The wild-type p53 levels in ZR75.1 and MCF-7 cells increased markedly after transfection with Apo2 (Figure 4A), whereas the inherently high levels of mutant p53 protein in MDA-MB-231 cells did not vary with the treatment (Figure 4A). The upregulation of p53 seemed to reflect protein stabilisation rather than enhanced gene transcription, as no increase in p53 mRNA levels was detected by RT–PCR in Apollon downregulated cells (Figure 4B).

To explore in more detail the molecular mechanisms of the induction of apoptosis by Apollon knockdown, we examined the expression of proteins involved in the mitochondrial pathway. Transfection with Apo2 induced the accumulation of Bax and Bad proteins and a remarkable release of the AIF from mitochondria into the cytosol in cells with wild-type p53, but not in cells expressing mutant p53 (Figure 4C). In addition, although to a variable extent, an increase in the total Smac/DIABLO was observed in all cell lines (Figure 4C). The cytochrome *c* release from mitochondria was also determined by ELISA after subcellular fractionation (Figure 4D). The results indicated that Apollon knockdown caused an enhancement of the cytochrome *c* cytosolic fraction in the two cell lines expressing wild-type p53. Specifically, the release of cytochrome *c* was 12- and 9-fold higher in ZR75.1 and MCF-7 cells treated with Apo2, respectively, than in cells transfected with the control siRNA (Figure 4D). By contrast, in MDA-MB-231 cells bearing mutant p53, Apollon knockdown did not cause any cytochrome *c* release (Figure 4D). Such findings were corroborated by western blot data obtained on isolated mitochondrial and cytosolic fractions, which indicated a marked decrease in the amount of cytochrome *c* remaining in mitochondria in ZR75.1 and MCF-7 cells, but not in MDA-MB-231 cells (Figure 4E).

### Wild-type p53 and caspase-3 seem to be necessary for the execution of apoptosis induced by Apollon knockdown

**Figure 5:**
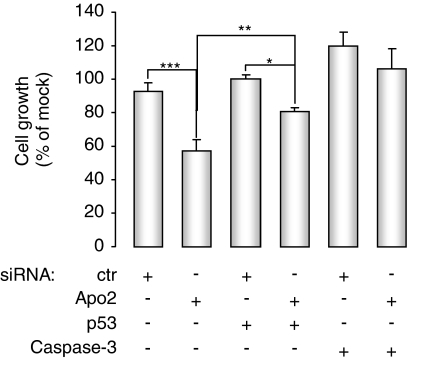
Effects of the combined downregulation of Apollon and p53 (or caspase-3) in ZR75.1 cells. *In vitro* growth of ZR75.1 cells 72 h after transfection with ctr (10 nM) and Apo2 (10 nM) siRNAs alone or in combination with p53 (25 nM) and caspase-3 (25 nM) siRNAs. Data are expressed as percentage values of growth in cells transfected with different siRNAs compared with mock control, and represent the mean values±s.d. of at least three independent experiments. ^*^P<0.05, ^**^P<0.01 and ^***^P<0.001. siRNA=small interfering RNA.

To determine to what extent wild-type p53 influences the induction of apoptosis consequent on Apollon downregulation, we knocked down p53 in ZR75.1 cells by RNAi. Pre-incubation of cells with p53-specific siRNA resulted in a partial rescue of cell growth inhibition (Figure 5) and a marked reduction of apoptosis (Figure 3). These observations, which are consistent with the modest level of apoptosis observed in Apo2-transfected MDA-MB-231 cells, bearing mutant p53 (Figure 3), suggest that the wild-type p53 protein is a major player in the induction of programmed cell death by Apollon knockdown.

It is noteworthy that apoptosis induction resulting from Apollon silencing was only reduced partially, but not abrogated completely after p53 knockdown, suggesting that Apollon downregulation can also interfere with caspase-3 activity in a p53-independent manner. To address this hypothesis, we tested the effect of the simultaneous knockdown of caspase-3 and Apollon in ZR75.1 cells. We found that the siRNA-mediated inhibition of caspase-3 catalytic activity resulted in the complete rescue of cell growth inhibition induced by Apollon silencing (Figure 5), as well as in the total abrogation of apoptosis although in the presence of activated caspase-9 (Figure 3). These findings are reminiscent of what we observed in MCF-7 cells transfected with Apo2 (Figures 2 and 3), in which induction of apoptosis was prevented by the absence of caspase-3 also in the presence of caspase-9 activation.

## Discussion

In this study, we evaluated the effects of RNAi-mediated Apollon gene silencing in human breast cancer cell lines. Transfection with a siRNA able to consistently induce an almost complete abrogation of Apollon protein expression in all cell lines, variably influenced cell growth as a function of *TP53* gene status of the tumour model and the availability of a functional caspase-3. Specifically, in the wild-type-p53 ZR75.1 cells, Apollon silencing caused a marked impairment of cell growth, which was paralleled by upregulation of p53, activation of the mitochondrial apoptotic pathway including Bax upregulation, release of cytochrome *c* and AIF from mitochondria, and consequent enhancement of caspase-9 and caspase-3 activity. The activation of caspase-8 was also observed. Likewise, p53 upregulation and activation of the mitochondrial pathway was also observed in the wild-type-p53 MCF-7 cell line. However, as these cells lack caspase-3 (Janicke *et al*, 1998; Yang *et al*, 1998), no cell growth impairment and no evidence of caspase-8 activation or apoptosis induction was found in these cells.

**Figure 6:**
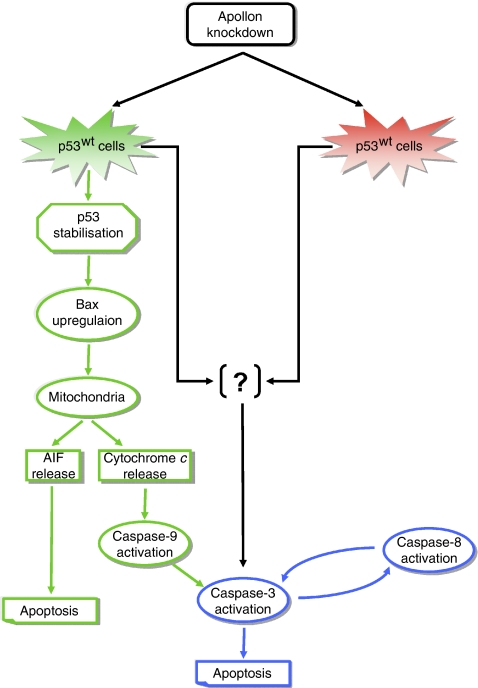
Proposed model of apoptosis induction by Apollon knockdown in breast cancer cells as a function of the *TP53* gene status. In wild-type p53 expressing cells, p53 stabilisation results in Bax upregulation and translocation to mitochondria with the consequent induction of cytochrome *c* and AIF release. Cytochrome *c* in turn activates caspase-9 and caspase-3, whereas AIF promotes caspase-independent apoptosis. In these cells, as well as in cells carrying mutant p53, additional p53-independent caspase-3 activation seems to occur. AIF=apoptosis inducing factor; siRNA=small interfering RNA.

Although to a lesser extent, compared with ZR75.1 cells, growth retardation, as well as apoptosis induction as a consequence of enhanced caspase-3 and caspase-8 activity were also observed in mutant-p53 MDA-MB-231 cells. The latter finding, together with the evidence that concomitant downregulation of p53 by RNAi only partially rescued cell growth inhibition and reduced, but not abrogated completely, apoptosis induced by Apollon knockdown in ZR75.1 cells, led us to hypothesise that Apollon downregulation can also directly or indirectly activate caspase-3 by a p53-independent signal (Figure 6). Experimental support for this hypothesis comes from experiments of simultaneous silencing of caspase-3 and Apollon in ZR75.1 cells, which showed that the abrogation of the enzyme's catalytic activity resulted in a complete rescue of cell growth inhibition and total abrogation of apoptosis induced by Apollon silencing.

Our findings corroborate and extend earlier observations by Ren *et al* (2005) indicating that inactivation of Bruce, the murine homologue of Apollon, through deletion of the C-terminal UBC domain, induced apoptosis in mouse embryonic fibroblasts through upregulation and nuclear localisation of wild-type p53, activation of the mitochondrial pathway of apoptosis and the consequent activation of caspase-9 and caspase-3. The same authors showed that the RNAi-mediated Apollon silencing in the p53^+/+^ human lung cancer cell line H460, also induced a strong apoptotic response as a consequence of p53-dependent activation of the mitochondrial pathway. Moreover, simultaneous silencing of Apollon and p53 in these cells induced only a partial rescue of cell viability and a limited decrease in caspase-3 activation (Ren *et al*, 2005). Both our studies and Ren's studies (Ren *et al*, 2005) point to a central role of p53 as a downstream effector of Apollon/Bruce. Although it is not known how Apollon knockdown causes p53 stabilisation, no interference with *p53* gene transcription was reported in either study. Considering that Apollon is itself an E2/E3 ubiquitin ligase, it could act similarly to Mdm2, Pirh2 or COP1, directly catalysing p53 ubiquitination and proteasome degradation (Leng *et al*, 2003; Dornan *et al*, 2004).

The possibility that Apollon knockdown enhances caspase-3 catalytic activity in a direct way is supported by the observation of Bartke *et al* (2004), who reported that Apollon interacts strongly with caspase-3 and inhibits its activation in a cell-free extract of 293T adenovirus-transformed human embryo kidney cells. However, such findings were not confirmed by Qiu and Goldberg (2005) in the same experimental model. An alternative possibility is that increased caspase-3 activity could be the result of caspase-8 activation after Apollon knockdown. In this context, it has been reported recently that apoptosis induction after transduction of HT-1080 human fibrosarcoma cells with an oncolytic adenovirus, expressing a short hairpin RNA against Apollon, was mediated by the proteolytic activation of caspase-8 and caspase-3 (Chu *et al*, 2008).

In our study, we observed caspase-8 activation after Apollon knockdown only in ZR75.1 and MDA-MB-231, but not in the caspase-3-deficient MCF-7 cell line and in ZR75.1 cells after siRNA-mediated caspase-3 silencing. Such evidence would suggest a possible alternative interpretation of the results, in that the active form of caspase-3 binds pro-caspase-8 and activates it (Sartorius *et al*, 2001; Wajant, 2003), which in turn can activate caspase-3 (Figure 6). Somewhat surprisingly, the results we obtained in the mutant-p53 MDA-MB-231 cells did not show any caspase-9 activation after Apollon knockdown. In fact, earlier observations obtained by Qiu and Goldberg (2005) in cell-free systems indicated that immunopurified Apollon binds *in vitro* to pro-caspase-9 and caspase-9 (although in a different study, only a weak binding of Apollon to caspase-9 was detected (Bartke *et al*, 2004)), and inhibits the enzyme's catalytic activity (Qiu and Goldberg, 2005). In addition, in 293T cells ectopically expressing Apollon, it was shown that the protein ubiquitinated and facilitated the degradation of caspase-9 (Hao *et al*, 2004). However, as all this evidence has been derived from cell-free systems or living cells forced to express Apollon, it is difficult to compare it with the findings we obtained in cells that were not manipulated to increase their Apollon abundance. It is worthy to note that no published data dealing with the interference of Apollon/Bruce with caspases on cells expressing mutant p53 are currently available.

Overall, our results indicate that the Apollon gene silencing causes growth impairment in breast cancer cells carrying a wild-type or mutant p53 gene, mainly as a consequence of apoptosis induction, and identifies p53 stabilisation and caspase-3 activation as major events that concur to determine the apoptotic response. These data also suggest the opportunity to conduct a survey of the tumour types that express Apollon, and to further proceed with the validation of Apollon as a potential new therapeutic target for malignancies, in which it is overexpressed. However, considering that in our study, a total decline of breast cancer cell growth was not observed despite an almost complete abrogation of the Apollon expression, targeting Apollon alone could be insufficient for the effective treatment of tumours overexpressing it. As earlier evidence points to the possibility of improving the activity of specific anticancer drugs through Apollon knockdown (Chen *et al*, 1999; Chu *et al*, 2008), such a chemosensitising effect could be exploited for the design of more effective therapies with Apollon inhibitors combined with anticancer drugs to be used in the clinic, after a preclinical validation of the most appropriate ways of combining the different agents in chemoresistant tumour models. However, as the presence of wild-type p53 and ‘normal’ caspase-3 expression seem to be the major determinants of the anti-proliferative effects consequent to Apollon downregulation, the possible induction of toxicity in normal tissues should be verified carefully in animal models.

## Figures and Tables

**Table 1:** Sequences of control siRNA and siRNAs targeting Apollon mRNA

**Name**	**Sequence**
Ctr	CAAUCUGUAGAUAUAGUGAdTdT dTdTGUUAGACAUCUAUAUCACU
Apo1	AGAAAUUGACCUUGAGUUAdTdT dTdTUCUUUAACUGGAACUCAAU
Apo2	GCAGUACAUGGUAUGAUUAdTdT dTdTCGUCAUGUACCAUACUAAU
Apo3	AGACUGCUGAGAUAGUUUAdTdT dTdTUCUGACGACUCUAUCAAAU
Apo4	UGAAUCCUGCACUAAUUCAdTdT dTdTACUUAGGACGUGAUUAAGU

siRNA=small interfering RNA
